# Learning technology for Ayurveda with pedagogically designed virtual patient case simulations

**DOI:** 10.3389/fmed.2025.1537047

**Published:** 2025-06-20

**Authors:** Prema Nedungadi, Rammanohar Puthiyedath, Romita Jinachandran, Shyamasundaran Kulangara, Sushma Naranappa Salethoor, Mandip Goyal, Nagarajan Chockan, Anup Thakur, Raghu Raman

**Affiliations:** ^1^School of Computing, Amrita Vishwa Vidyapeetham, Amritapuri, India; ^2^Amrita CREATE, Amrita Vishwa Vidyapeetham, Amritapuri, India; ^3^Amrita School of Ayurveda, Amrita Vishwa Vidyapeetham, Amritapuri, India; ^4^Institute of Teaching and Research in Ayurveda (ITRA), Jamnagar, India; ^5^Amrita School of Business, Amrita Vishwa Vidyapeetham, Amritapuri, India

**Keywords:** complementary and alternative medicine, learning technology, TCAM, virtual patients, Ayurveda, technology-enhanced learning, SDG3: good health and wellbeing, SDG4 quality education

## Abstract

**Introduction:**

Ayurveda education in India has seen limited integration of learning technology, leading to significant gaps in clinical skill development. This study introduces AyurSIM, a virtual patient simulation platform designed with interactive virtual cases replicating real-world scenarios to address this gap, offering structured educational modules from patient examination through follow-up care, incorporating Ayurveda protocols and a comprehensive database of disease patterns.

**Methods:**

AyurSIM was developed using pedagogical frameworks building on experiential learning, problem-solving tasks, and reflective practice. The platform enables the creation of regionally adapted virtual cases, aligning with diverse Ayurvedic practices while maintaining a structured protocol. Usability was evaluated using the validated System Usability Scale (SUS) among 210 diverse participants during training workshops.

**Results:**

The platform achieved a “good” SUS score of 77.08. Participants commended the intuitive interface, immersive learning experience, and educational value. Suggested improvements included integrating AR/VR technologies, expanding clinical content across disciplines, and multilingual support.

**Discussion:**

AyurSIM effectively bridges traditional Ayurveda education with modern methodologies, providing culturally responsive, practical learning experiences. The findings suggest that the blended pedagogical approach provides a robust framework for Ayurveda education. The ability to simulate realistic virtual patient clinical scenarios in a structured manner underscores the platform’s value in preparing students for real-world practice.

## 1 Introduction

Ayurveda, a traditional, complementary and alternative medicine system, originated from the Indian subcontinent and is one of the oldest medicinal systems (over 3,000 years). Ayurveda focuses on balancing the mind and body through the combination of internal medications, external therapies, dietary rigor, yoga, and lifestyle modifications (1, 2). The Ministry of AYUSH in India is responsible for promoting, developing, and overseeing traditional medicine systems such as Ayurveda, Yoga, Naturopathy, Unani, Siddha, and Homeopathy, with the aim of improving healthcare and supporting overall wellness in the country. India has over 450 Ayurveda colleges that offer higher education programs, such as the Bachelor of Ayurvedic Medicine and Surgery (BAMS) programs and Doctor of Medicine in Ayurveda (MD) programs. The programs currently lack relevant technology-enhanced learning platforms to support student learning outcomes. AyurSIM, a virtual patient case simulation platform designed for Ayurveda undergraduate education, aims to bridge this gap with emerging technology.

Clinical simulations are effective tools for preparing students for real-world clinical scenarios, enhancing their clinical skills and decision-making processes (3). Learning technologies and simulated clinical exercises afford immersive and interactive ambiance, which are crucial for comprehension and clinical practice (4).

Interactive environments in other domains, such as simulation labs for high school experiments, simulated robotics (5), synthetic laboratories for engineering education (6), digital laboratories for additive manufacturing (7), and multilingual learning platforms for digitally famished village-based education, have been successfully implemented (8). A formative assessment-driven student-facing analytics dashboard notably doubled task completion rates in remote lab settings (9), whereas interactive, personalized health monitoring systems were used for training and support of community health workers in rural areas (10). A simulated, cost-effective medical simulation platform was strategically designed to augment conventional teaching practices and address routine-care and advanced-care medical scenarios (11).

Graduates of programs in the practice of Ayurveda are required to possess a foundational understanding of the discipline and its integration with other scientific and clinical faculties (12). Traditional pedagogical methods in medicine strive to sustain students’ attention, influencing their comprehension and acquisition of practical skills (13). Traditional complementary and alternative medicine (TCAM) uses technology that spans diverse clinical uses, such as anxiety disorders (14, 15), pain management (16, 17), simulated optometry (18), and advanced medical education (19, 20). The technological implementation of virtual reality (VR) in educational settings, along with advanced metaverse technologies, offers a dynamic platform for the delivery of immersive educational experiences tailored to distinctive learning encounters (21). However, the integration of virtual reality in resource-limited settings is impaired by a fragmented technical infrastructure and cost barriers (22). Simulation-based training has been widely recognized for its favorable impact on learning outcomes in medical education (23, 24).

The relevance of simulation in medical education is further highlighted by the global acknowledgment of gaps in healthcare quality and safety (25). The effectiveness of simulation-based medical education has been substantiated through meta-analysis (26). However, the potential for immersive technologies for Ayurvedic higher education remains untapped, presenting opportunities to enhance TCAM education through learning technologies.

Fidelity, albeit crucial in the development of simulations for education, is inadequate to guarantee preferred learning outcomes (27). Simulation-based learning has emerged as a pivotal component of medical education, offering hands-on experiential learning (28). Specifically, digital tools in Ayurvedic education can facilitate a deeper understanding of intricate concepts. These tools have the potential to bridge gaps between venerable traditional knowledge and modern educational practices. The impact of COVID-19 on the education system has been palpably significant. Persistent uncertainties in the resumption of regular classes have bolstered education delivery through online platforms (29). Disruption of traditional teaching methods has accelerated the incorporation of virtual learning environments into higher education, with the aim of achieving sustainable development goals 4 (quality education) (30, 31).

A cross-sectional study on the adoption of learning technologies within complementary and alternative medical education underscored the preference among students for blended learning environments, illustrative multimedia, interactive tools, and simulation-based learning to enhance bona fide skills (32). The unification of instructional technology in TCAM education remains at a nascent stage. Prevailing TCAM education includes online courses and modules for wider access to perceptive skill development, case studies on interactive learning experiences, and mobile apps. A study recommended global educational initiatives in complementary and alternative medical education, such as Ayurveda, with immersive learning based on modern pedagogical approaches (33).

The regional diversity in Ayurveda practices arises from geographical, cultural, and climatic differences across India, which affect the choice of medicinal herbs and treatment methods (43, 44). This diversity complicates the creation of clinical simulations. Addressing the multifaceted challenges of developing clinical case simulations for Ayurveda includes the creation of adaptive simulations that accommodate various regional practices, such as the integration of diverse protocols, treatment methods, and cultural contexts. This study investigates two key research questions that guide the development and evaluation of learning platforms:

RQ1: What theoretical frameworks support the design of a learning system specific to complementary and alternative medicine education, such as Ayurveda?

RQ2: How do learners perceive the user interface and usability of the system?

## 2 Theoretical framework

Experiential learning plays a fundamental role in the educational paradigm with hands-on experiences that closely replicate real-life scenarios, deepening students’ assimilation of the subject matter. Experiential learning bridges theoretical concepts with practical applications, reinforcing learning outcomes in real-world contexts, enhancing student engagement, and improving retention of knowledge (34).

Multimedia learning is utilized in modules that focus on patient diagnosis and inference of symptoms. The engagement of multiple senses through visual and auditory multimedia enriches learning, increasing the accessibility and retention of complex concepts. A previous study demonstrated the propriety of multimedia aids in diverse formats (35). Simulation-based learning is well documented for its effectiveness in medical education as a springboard for the application of theoretical knowledge in controlled, risk-free environments (36).

The focus on problem solving in pedagogic modules fosters critical thinking and decision-making skills. Accentuated analytical and practical problem solving is imperative for overcoming the complexities of clinical practice. Reflective practice promotes self-reflection on decisions and actions taken, fostering a deeper understanding of ongoing self-improvement. Reflective practice enables learners to evaluate their learning process and outcomes critically, promoting adaptability and lifelong learning in professional settings (37, 38).

Finally, the case study method is useful because of its ability to simulate real-life challenges, enabling students to understand situations (39). In the follow-up analysis modules, the case study method presents learners with complex scenarios requiring in-depth analysis and assessment. This method is instrumental in the application of theoretical knowledge to real-world situations.

The integration of pragmatic state-of-the-art pedagogical strategies into learning technology has the potential to enhance the learning experience through alignment with Ayurveda’s holistic perceptive approach. Experiential learning mirrors Ayurveda’s practical methods of patient examination and encourages students to develop hands-on diagnostic and treatment skills essential for restoring the natural balance within a patient’s body. Multidimensional learning complements Ayurveda’s multisensory techniques, embellishing students’ unmitigated comprehension of subject matter. Simulation-driven learning affords a risk-free platform for the exploration of Ayurvedic treatment, stimulating students’ ability to experiment with personalized treatment plans devoid of aggravated repercussions in the real world. The focus on problem-solving and reflective practice cultivates critical thinking, and self-reflection is exigent in the nuanced discipline of Ayurveda. Case study methods drive students into immersive, complex scenarios, bridging theoretical knowledge with practical application.

RQ1 examines how incorporating pedagogical strategies contributes to a comprehensive learning framework for Ayurveda education. These strategies foster a learning environment based on established techniques that prepare students with well-rounded training to take on realistic challenges in the Ayurveda domain.

## 3 Methodology

This study employed a mixed-method approach to design, implement, and evaluate AyurSIM, a virtual patient simulation platform tailored for Ayurveda education. The development of the platform was guided by pedagogical frameworks, including experiential learning, multimedia learning, problem-solving, and reflective practice. These principles were integrated into interactive modules covering patient examination, diagnosis, treatment prescription, and follow-up.

The architecture of AyurSIM was built via a modular design framework, employing Django and React.js for scalability and interactive user interfaces. The platform incorporated Ayurvedic principles such as Prakriti (somatic constitution) and Doshas (functional regulators) (41), using a rule-based engine and data analytics tools to create regionally adaptive clinical simulations. The interactive modules featured multimedia content, including audio, video, 2D/3D animations, and quizzes, to enhance learner engagement.

The usability of the platform was evaluated through workshops involving 210 participants, including students, educators, and practitioners. The system usability scale (SUS) was used for quantitative analysis, yielding an average score of 77.08, which was classified as “good.” Qualitative feedback was collected to identify strengths and areas for improvement, such as the integration of AR/VR technologies and multilingual support.

This structured approach enabled the development of an immersive, scalable, and culturally responsive learning tool, bridging traditional Ayurvedic education with modern technological advancements.

### 3.1 AyurSIM framework and architecture

**Figure 1:**
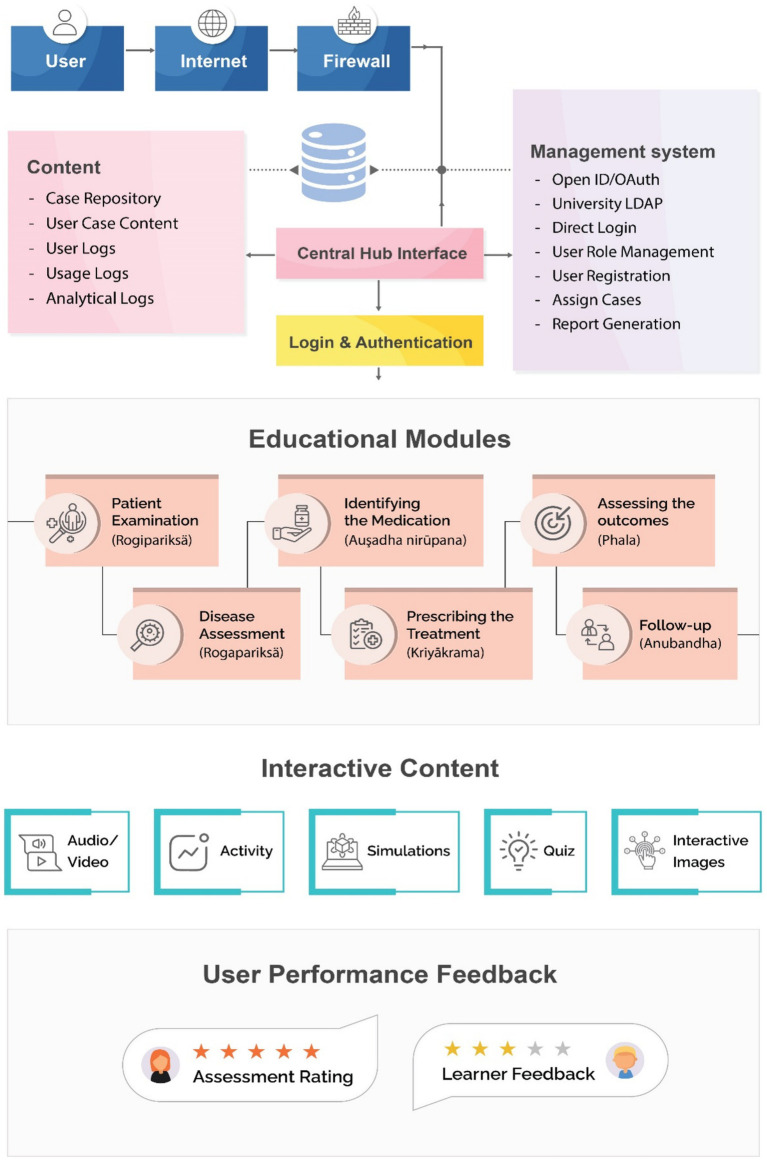
System architecture.

Figure 1 illustrates the architecture of the learning platform. The design of the virtual clinical simulations is inspired by the widely used method of documentation covering notes on the subjective, objective, assessment, planning, intervention, evaluation and revision (SOAPIER) components. The framework builds clinical simulations incorporating Ayurveda’s intricate principles, such as the somatic constitution (Prakriti) and the body’s functional regulators (Doshas) (Annexure Table A1). The platform uses Django for rapid development and modular design, applying the model–view–controller (MVC) pattern for scalability and efficient updates. The front end employs React.js, with the Django Rest Framework handling APIs and MySQL for data storage. The platform integrates libraries such as Pandas for analytics and OpenCV for multimedia processing.

The platform connects to external systems through restful APIs. A rule-based engine supported by Scikit-learn drives context-sensitive recommendations aligned with Ayurvedic protocols.

For developers, the system enables the rapid development of clinical cases, allowing for the creation of immersive educational experiences. The platform allows for region-specific variations of virtual patients who mirror the demographic, cultural, and health profiles prevalent across India’s diverse regions.

For learners, the interactive educational modules include examination of the virtual patient, assessment of the disease, identification of the medications, prescription of the treatment, assessment of the outcomes, and follow-up. The interactive content includes audio, video, animation, interactive 2D and 3D media, simulations, educational activities, and assessments. Reporting and visualization are available for learners, educators, and institutions to understand the learning pathway.

The platform is available online for free access, requires user registration, and can be accessed at: https://ayursim.amritacreate.org/.

### 3.2 Educational modules and pedagogy

#### 3.2.1 Educational modules

**Figure 2:**
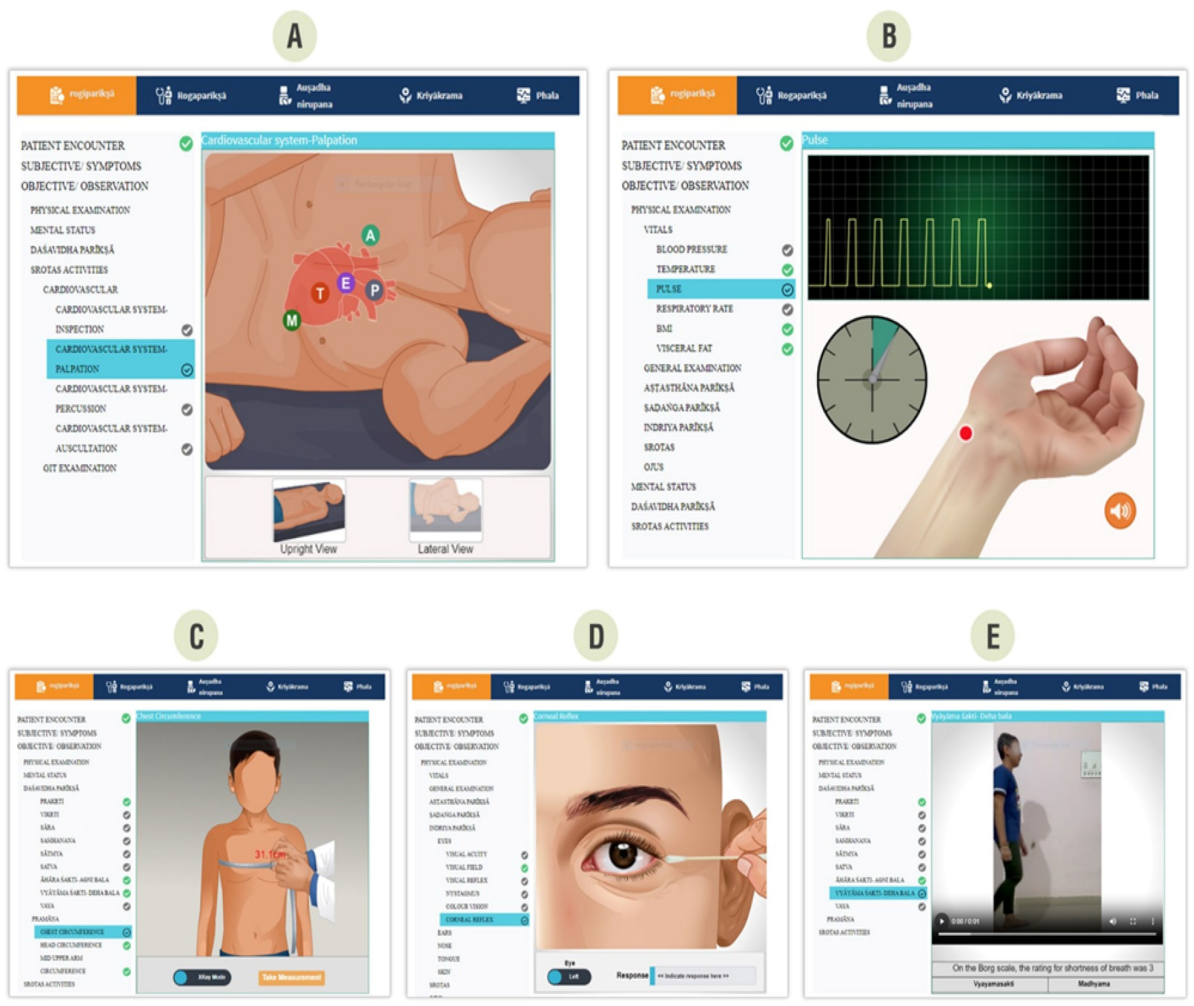
Sample user interface of clinical case.

The learner uses the virtual patients with the following educational modules in a sequential manner. Figure 2 shows the user interface design for examining the virtual patient.

##### 3.2.1.1 Virtual patient examination (*Rogipariksha*)

This module offers a comprehensive examination of the patient and compiles all relevant clinical findings to facilitate case assessment and diagnosis. The subjective submodule comprises two key elements: listening to the patient and history taking. The module narrates the subjective symptoms experienced by the patient. It starts with a virtual patient encounter that offers a visualization of the patient’s condition through video recordings, followed by interactive activities for the learner to identify the signs and symptoms currently exhibited by the virtual patient. The objective of the observation module is to capture the findings made by the learner during the clinical assessment of the virtual patient, such as abnormal heart sounds or a palpable lump in the armpit that the patient may not be aware of.

##### 3.2.1.2 Patient assessment (*Rogapariksha*)

Unlike the preceding examination module, the module focuses on the diagnosis of diseases associated with each patient. The system looks at symptoms, disease patterns, and pathological processes for a comprehensive assessment of clinical findings to arrive at a specific diagnosis.

Identifying the medications (*Aushadha Nirupana*): The system enables the student to formulate a tailored medication using the results obtained from the previous modules and data-driven algorithms.

##### 3.2.1.3 Prescribing the treatment (*Kriyakrama*)

This module allows the development of treatment plans and prescriptions via a rule-based engine. This enables the student to incorporate aspects such as counseling, lifestyle, and diet in the final prescription, providing a holistic approach to patient care.

##### 3.2.1.4 Assessing the outcomes (*Phala or Siddhivimarsha*)

This module is for the postmedication phase and employs reporting tools to articulate the results obtained during the earlier follow-up visits. This includes targeted symptoms, patient responses, and provisions for revising prescriptions.

##### 3.2.1.5 Follow-up (*Anubandha*)

The module offers a consolidated report of further follow-up visits and an executive summary of the case. This is useful for demonstrating long-term outcomes to students.

Data analytics and visualization tools synthesize information into an accessible format, supporting the analysis of student learning.

## 4 Discussion and usability findings

### 4.1 RQ1: education strategies in AyurSIM

The AyurSIM platform integrates a range of contemporary educational strategies, such as experiential learning, multimedia learning, problem-solving, and reflective practice (Table 1).

**Table 1:** Mapping educational modules to pedagogy.

Ayurveda module	Learning theory	Virtual patient interaction
Subjective Virtual patient encounter	Multimedia learning	Shows the video recordings of the patient explaining the signs and symptoms they currently exhibit.
Subjective Symptoms	Multimedia learning	Involves interacting with the virtual patient, playing audio recordings of patient encounters, and history taking.
Objective Observation	Experiential learning	Interactive activities based on each case, simulating real environment functionalities with a Simulation Engine.
Assessment Virtual patient examination	Experiential learning	Focuses on examining the virtual patient for diagnosing and treating diseases using different methods.
Assessment Diagnosis of Disease	Problem-solving	Provides a complete picture using Ayurvedic algorithms for assessing variables such as Ama, Dosha, and Sthana, utilizing Ayurveda protocols and a database of disease patterns.
Plan Identifying medication Prescribing the treatment	Problem-solving	Concludes the results from other categories and helps formulate the final prescription, aided by a rules-based engine.
Assessing outcomes	Reflective practice	Review the results obtained after medication during the follow-up visit for the case with reporting tools.
Subjective Virtual patient encounter	Multimedia learning	Shows the video recordings of the patient explaining the signs and symptoms they currently exhibit.

Experiential learning is at the core, allowing students to engage in realistic patient scenarios through simulated interactions. Multimedia learning, supported by video and audio recordings, enriches the learning experience by engaging multiple senses and making complex Ayurvedic concepts more accessible. Interactive simulations provide a controlled environment where students can experiment without risk, supported by robust simulation engines that mimic real-world clinical settings.

The modules for virtual patient examination and for identifying medications focus on enhancing problem-solving and decision-making skills, offering scenarios that challenge students in the application of diagnostic algorithms and treatment protocols. Reflective practice is embedded within the module that assesses the outcomes, encouraging learners to critically analyze their clinical decisions and outcomes and promoting deeper understanding and ongoing improvement. The platform’s instructional design is further strengthened by providing students with a consolidated report of each follow-up visit’s details and an executive summary via data analytics and visualization tools.

While we employ experiential learning broadly, AyurSIM modules align conceptually with Kolb’s four-stage experiential learning cycle. Specifically, learners engage in concrete experience through interactive case simulations, followed by reflective observation during feedback. Abstract Conceptualization occurs through video explanations, culminating in active experimentation via repeated interactive sessions.

### 4.2 RQ2: system usability

The platform was evaluated during 2 workshops via the widely used System Usability Scale (SUS) (40). The survey respondents included 210 participants, including students, educators, and practitioners. SUS employs a 10-item questionnaire with responses ranging from “Strongly Agree” to “Strongly Disagree,” as outlined in Annexure Table A2. The survey comprises an equal number of positively and negatively worded statements. Scoring is inversely applied to the negative items.

**Figure 3:**
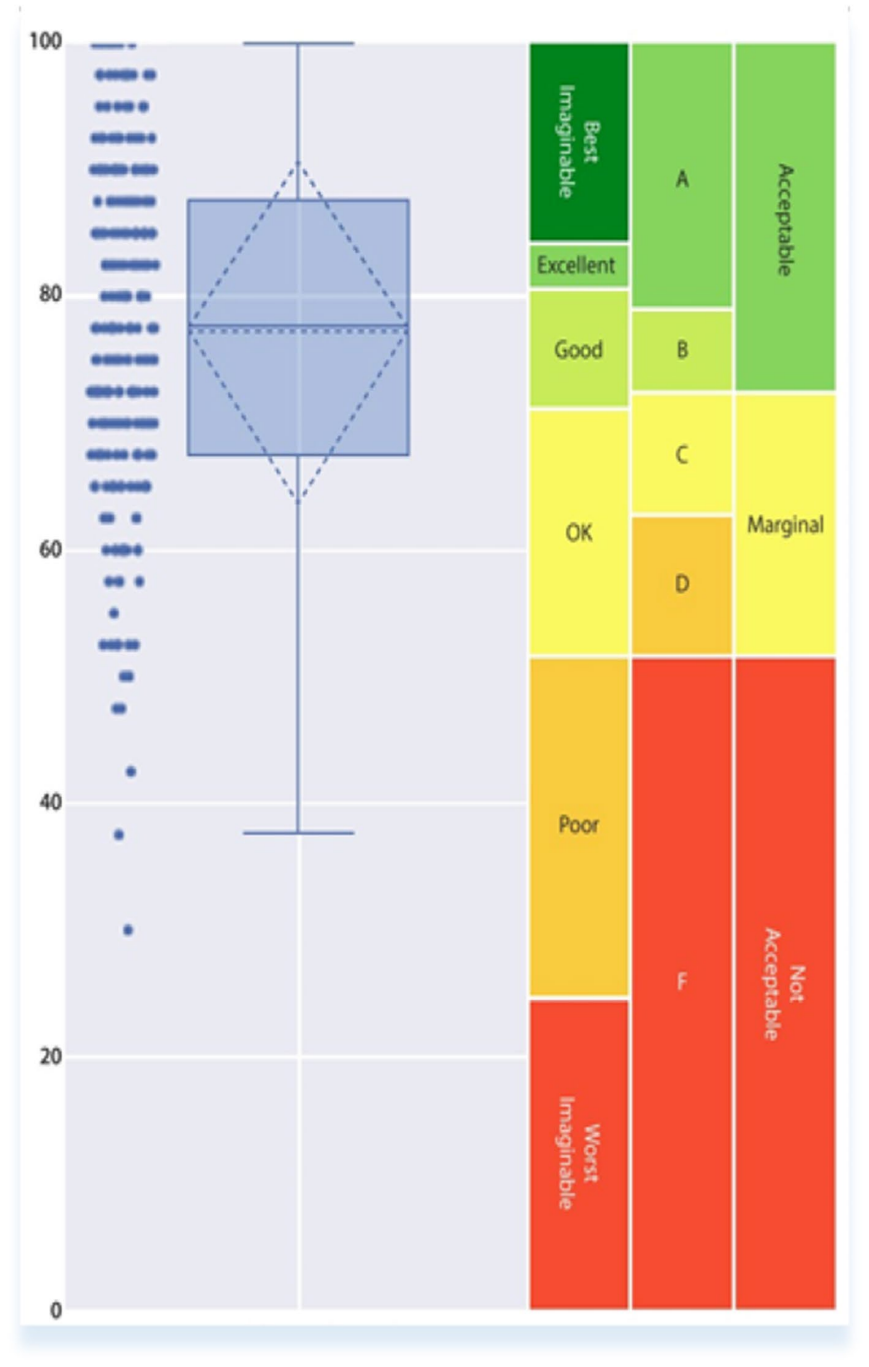
Boxplot system usability scale study score.

Figure 3 shows a box plot with the mean and standard deviation alongside the raw data points and SUS contextualization scales. The box plot illustrates a clustering of scores, mainly within the upper 70s to mid-80s, indicating a notably high level of usability for the system. The average SUS score of 77.08, with a standard deviation of 13.65, which is categorized as “good” in SUS evaluations, indicates a high level of usability.

#### 4.2.1 Qualitative feedback

The qualitative feedback highlights the platform’s user-friendly interface and the integration of simulations with real-world scenarios. Users noted intuitive navigation and appreciated the structured learning paths that guided students through diagnostic and treatment processes. Interactive multimedia components, such as video-based patient interactions and audio modules, are frequently praised for enhancing user engagement and comprehension.

Users consistently reported that AyurSIM bridges the gap between traditional Ayurvedic education and modern technology by providing practical, hands-on learning in a controlled environment. One user summarized the platform’s impact, stating, “AyurSIM’s simulations bring theoretical knowledge to life, making learning engaging and applicable to real clinical settings.

User feedback reinforced the educational value of AyurSIM, particularly its comprehensive coverage of Ayurvedic practices and intuitive interface. The interactive elements, including quizzes and multimedia simulations, were well received to enhance engagement and practical learning.

#### 4.2.2 Interactive learning experience

The platform provides comprehensive Ayurvedic education through practical simulations and case studies. Users find it effective in bridging theoretical knowledge with practical application, enhancing their learning experience. One user noted, “The case studies and simulations bring theories to life, offering a hands-on learning experience that is both engaging and informative.” The intuitive interface facilitates easy navigation, making it accessible even for those unfamiliar with digital platforms. As one user mentioned, “The layout is intuitive and straightforward, making it easy for even the least tech-savvy users to navigate”.

The platform leverages pedagogy in technology to create an engaging educational environment for Ayurvedic studies. The interactive simulations allow users to apply theoretical knowledge in virtual scenarios, enhancing their understanding. One user expressed, “The interactive simulations on AyurSIM have been a game changer for my learning, allowing me to apply what I have learned in a virtual setting.” The platform’s performance and responsive design contribute to a positive learning environment, minimizing technical disruptions.

#### 4.2.3 Suggestions for improvement

However, feedback also identified areas for future improvement, such as expanding language options and integrating advanced technologies such as AR and VR for more immersive experiences. One user stated, “The prospect of VR and AR integration is thrilling, promising a future where we can ‘experience’ Ayurvedic practices in a virtual space”.

While the platform currently offers cases in nine domains, viz. Agada Tantra—Dept. of Forensic Medicine and Toxicology, Kayachikitsa—Dept. of General Medicine, Kaumarabhritya—Dept. of Pediatrics, Manasaroga—Dept. of Psychiatry, Panchakarma—Dept. of Major Therapeutic Procedures, Prasuti Tantra & Stree Roga—Dept. of Obstetrics and Gynecology, Shalakya Tantra (Dental and ENT)—Dept. of Dental and ENT, Shalakya Tantra Netra (Eye)—Ophthalmology and Shalya Tantra—Dept. of Surgery, users have requested additional topics, including modern diagnostics, gynecology, obstetrics, and emergency medicine. Users also suggested incorporating virtual and augmented reality cases.

A key suggestion for improvement is to include more local languages, such as Hindi, to align with Ayurveda’s practice in native languages and broaden accessibility. A user suggested that “incorporating more languages will make AyurSIM accessible to a wider audience, breaking down language barriers in Ayurvedic education”.

### 4.3 Implications for theory and practice

The incorporation of learning theories such as experiential learning, multimedia learning, problem-solving, and reflective practice can increase engagement and can help prepare practitioners for the complexities of traditional, complementary, and alternative medicine.

The integration of virtual simulations creates a more interactive and engaging learning environment, bridging the gap between theoretical knowledge and practical application. The successful integration of advanced technologies in Ayurveda demonstrates the potential for modern tools to enhance the educational practices of complementary and alternative medicine.

For practitioners, the platform offers a novel tool for continuous learning and professional development. By simulating a diverse range of scenarios, such systems can help practitioners prepare for various patient cases, enhancing adaptability and competence in real-world settings.

Global outreach and accessibility: The digital nature of AyurSIM enhances its accessibility, aiding in the global dissemination of Ayurvedic knowledge.

## 5 Limitations

While AyurSIM represents a significant advancement in pedagogical tools, certain limitations remain. The virtual simulations limit engagement with the physical aspects of Ayurvedic practice. While the platform allows for the incorporation of regional variations, the diversity of Ayurvedic practices across India is not fully captured owing to the scope of the current project. Much of Ayurveda practice is in regional languages, and limiting the platform to English can reduce access to rural students (42).

Despite limitations in engaging with the physical aspects of practice and fully capturing regional and physician-specific variations, interactive simulations and user-friendly interfaces provide valuable hands-on learning experiences.

There are several barriers to institutionalizing AyurSIM across the Ayurveda Colleges in India. Technological and infrastructure barriers include a lack of reliable internet, computers, or smart classrooms in rural areas. AyurSIM is not yet mandated by the National Commission for Indian System of Medicine (NCISM) for the BAMS curriculum, which may lead to reluctance to adopt AyurSIM at institutions. Senior faculty members may be hesitant to use digital tools for training.

These barriers can be overcome by the integration of AyurSIM into the NCISM-approved curriculum, the development of multilingual versions, and the inclusion of region-specific case studies, treatment protocols and training programs and workshops.

## 6 Conclusion

The learning platform offers comprehensive and practical experience through the integration of contemporary educational theories. The findings related to RQ1 suggest that the blended pedagogical approach adopted by AyurSIM provides a robust framework for Ayurveda education. The learning platform’s virtual patients, immersive environment, and comprehensive end-to-end support, from patient encounters to follow-up and reporting, have the potential to enhance learning outcomes for Ayurveda students. The ability to simulate realistic clinical scenarios in a structured manner underscores the platform’s value in preparing students for real-world practice.

Future developments in AyurSIM will focus on integrating AI and AR to provide personalized learning experiences and customize content for diverse regional practices. Research can assess educational impact through quantitative and qualitative methods, including pretests, posttests, and surveys. Feasibility studies explore the integration of VR and AR for immersive learning. Long-term studies monitor the platform’s impact on students’ performance and career development. Future work will focus on integrating AI-driven adaptive learning. This development aims to tailor educational content to individual learning needs, improving the overall effectiveness of Ayurveda education. The key areas of enhancement include the following:

- Adaptive learning algorithms with AI algorithms adjust simulation content and difficulty on the basis of real-time analysis of student performance, ensuring personalized learning pathways. The system can provide comparative treatment options, suggesting how different regional traditions may have different treatment plans.
- In the future, AI can automate learner feedback on the basis of diagnostic and therapeutic decisions made by the learner. An AI-enabled semantic search engine can link the case content to relevant references from classical texts. AI-enhanced instructor tools can be incorporated into a dashboard revealing learning analytics.
- Integrating NLP models in regional languages will enable more interactive simulations, providing instant feedback and guidance during virtual patient encounters.

## Data Availability

The raw data supporting the conclusions of this article will be made available by the authors, without undue reservation.
