# Supplementary material for: Learning technology for Ayurveda with pedagogically designed virtual patient case simulations
Source: Front Med (Lausanne). 2025 Jun 20;12:1537047. doi: 10.3389/fmed.2025.1537047 (PMC12226514; doi:10.3389/fmed.2025.1537047)
Supplement: Supplementary file 1 [file Table_1.docx]

# Supplementary Material for <https://www.frontiersin.org/journals/medicine/articles/10.3389/fmed.2025.1537047>

# Annexure 1

**Table A1. Mapping Sanskrit nomenclature to the learning system:**

| ***Prakriti*** | **Somatic constitution** |
| --- | --- |
| *Doshas* | Body's functional regulators |
| *Kayachikitsa* | General Medicine |
| *Panchakarma* | Biocleansing therapies |
| *Agada Tantra* | Toxicology |
| *Kaumarabhritya* | Pediatrics |
| *Streeroga & Prasuti Tantra* | Gynecology & Obstetrics |
| *Shalya Tantra* | Surgery |
| *Shalakya Tantra Netra Roga* | Specialization in treatment of Eye Diseases |
| *Shalakya Tantra* | Dentistry & ENT |
| *Manasa Roga* | Psychiatry |
| *Charakasamhita* | Ayurvedic treatise on General Medicine |
| *Susrutasamhita* | Ayurvedic treatise on Surgery |
| *Bhaisajyaratnavali* | Ayurvedic treatise on Treatments and Formulations |
| *Astangahridayam* | Ayurvedic treatise on eight clinical specialties of Ayurveda |
| *Rasashastra* | Specialization in Ayurveda that deals with herbo-mineral and metallic preparations |
| *Shirolepa* | An Ayurvedic therapy of applying medicated paste to the scalp |
| Pizhichil | An Ayurvedic therapy of application of oil and simultaneous massaging |
| *Siddha* system | An Indian system of medicine prevalent in Tamil Nadu |
| *Marma* | Vital points of the body |
| *Rogipariksha* | Examination of the patient |
| *Rogapariksha* | Assessment of the disease |
| *Aushadhanirupana* | Identifying the medications |
| *Kriyakrama* | Prescribing the treatment |
| *Phala*/*Siddhivimarsha* | Assessing the outcomes |
| *Anubandha* | Follow-up |

# Annexure 2

**Table A2.** SUS 10-item questionnaire

| ***No*** | ***Statements*** |
| --- | --- |
| **1** | I think that I would like to use AyurSIM often. |
| **2** | I found AyurSIM unnecessarily complex |
| **3** | I thought AyurSIM was easy to use. |
| **4** | I think that I would need the support of a technical person to be able to use AyurSIM. |
| **5** | I found the various functions of AyurSIM were well integrated (work well together). |
| **6** | I thought there was too much inconsistency in AyurSIM. |
| **7** | I think that most people would learn to use AyurSIM very quickly. |
| **8** | I found the ayusim very cumbersome to use (hard to use). |
| **9** | I felt very confident using AyurSIM. |
| **10** | I needed to learn a lot of things before I could get going with this AyurSIM. |
